# Systematic and quantitative mRNA expression analysis of TRP channel genes at the single trigeminal and dorsal root ganglion level in mouse

**DOI:** 10.1186/1471-2202-14-21

**Published:** 2013-02-14

**Authors:** Ine Vandewauw, Grzegorz Owsianik, Thomas Voets

**Affiliations:** 1 Laboratory of Ion Channel Research, Department of Cellular and Molecular Medicine and TRPLe (TRP Research Platform Leuven), KU Leuven, Leuven, Belgium

**Keywords:** Dorsal root ganglia, Trigeminal ganglia, TRP channels, Somatosensation, mRNA expression analysis, Sensory neurons

## Abstract

**Background:**

Somatosensory nerve fibres arising from cell bodies within the trigeminal ganglia (TG) in the head and from a string of dorsal root ganglia (DRG) located lateral to the spinal cord convey endogenous and environmental stimuli to the central nervous system. Although several members of the transient receptor potential (TRP) superfamily of cation channels have been implicated in somatosensation, the expression levels of TRP channel genes in the individual sensory ganglia have never been systematically studied.

**Results:**

Here, we used quantitative real-time PCR to analyse and compare mRNA expression of all TRP channels in TG and individual DRGs from 27 anatomically defined segments of the spinal cord of the mouse. At the mRNA level, 17 of the 28 TRP channel genes, TRPA1, TRPC1, TRPC3, TRPC4, TRPC5, TRPM2, TRPM3, TRPM4, TRPM5, TRPM6, TRPM7, TRPM8, TRPV1, TRPV2, TRPV4, TRPML1 and TRPP2, were detectable in every tested ganglion. Notably, four TRP channels, TRPC4, TRPM4, TRPM8 and TRPV1, showed statistically significant variation in mRNA levels between DRGs from different segments, suggesting ganglion-specific regulation of TRP channel gene expression. These ganglion-to-ganglion differences in TRP channel transcript levels may contribute to the variability in sensory responses in functional studies.

**Conclusions:**

We developed, compared and refined techniques to quantitatively analyse the relative mRNA expression of all TRP channel genes at the single ganglion level. This study also provides for the first time a comparative mRNA distribution profile in TG and DRG along the entire vertebral column for the mammalian TRP channel family.

## Background

Somatosensation describes the ability to detect a wide range of endogenous and environmental stimuli, such as temperature, mechanical forces, chemical stimuli and pain [1]. These stimuli are conveyed by sensory nerve fibres arising from cell bodies within the trigeminal ganglion (TG) in the head and within dorsal root ganglia (DRG), located lateral to the spinal cord in the vertebral column. To understand the mechanisms of somatosensation and nociception, it is important to identify the molecules that contribute to stimulus detection and promote excitation or sensitization of the primary sensory nerve fibres. In this context, a lot of attention has been focused on the cation-permeable ion channels of the transient receptor potential (TRP) family.

Due to their distinct activation mechanisms and biophysical properties, TRP channels are highly suited to function in sensory cells, either as receptors for environmental or endogenous stimuli or as molecular players in signal transduction cascades downstream of metabotropic receptors. As such, members of the TRP superfamily are involved in a variety of sensory processes, such as thermosensation, mechanosensation, osmosensation, olfaction, taste, vision and pain perception [2,3].

There are 28 mammalian TRP channels, which are subdivided into six subfamilies based on amino acid sequence homology: TRPA (ankyrin), TRPC (canonical), TRPM (melastatin), TRPML (mucolipin), TRPP (polycystin) and TRPV (vanilloid) [4]. To study their roles in the somatosensory system, it is important to know where and at what level they are expressed. Unfortunately, current expression studies in mammals are heterogeneous with regard to species, applied method, number of included tissues and cell types, and choice of analysed TRP channels. On the other hand, experiments in the past describing the expression profile of TRP channels in DRGs often gave diverging results [5-9]. It should be noted that there is a significant lab-to-lab or even a researcher-to-researcher variability in the segments of the vertebral column from which DRGs are isolated. Moreover, these differences are mostly not reported or taken into account [10-13]. We hypothesized that the reported discrepancy of TRP gene expression studies are, at least partly, due to variations in TRP gene expression depending on the segmental location of the DRG. We therefore set out to analyse the mRNA expression profile of all 28 mammalian TRP channels in TG and DRGs of each individual segment of the vertebral column of the mouse, thereby covering most of the sensory neurons innervating the skin, mucosa and internal organs. The obtained results form a firm basis for further studies on the relevance of TRP channels in sensory neurons in health and disease.

## Results

### Optimization of cDNA libraries for quantitative PCR analysis

RNA quantity and quality was assessed using the Experion Automated Electrophoresis System (BioRad) and revealed almost 2 orders of magnitude higher yield of total RNA extraction from TGs compared to that obtained from single DRGs (97.1 ± 20.1 ng/μl and 3.6 ± 0.3 ng/μl, respectively). In both cases the average quality of isolated total RNAs was relatively high reaching the RNA quality indicator (RQI) of about 8 (Additional file 1: Figure S1). Since amounts of total RNA from a single DRG of a defined segment of vertebral column were very low and in many cases expression levels of ion channel genes were rather poor, we fine-tuned the protocol for generation of cDNA libraries from all our samples. The SuperScript VILO cDNA Synthesis Kit (Life Technologies) was the most efficient in cDNA synthesis reaching from 1.5 to 4 units lower Ct values (Additional file 1: Figure S2 A). Moreover, all cDNA samples from DRGs were subjected to a preamplification step with the TaqMan PreAmp Master Mix Kit (Life Technologies) that further increased PCR detections as demonstrated by a reduction of Ct values by ~6 (Additional file 1: Figure S2 B).

### mRNA profiling of TRP genes in mouse TG and DRGs

**Figure 1:**
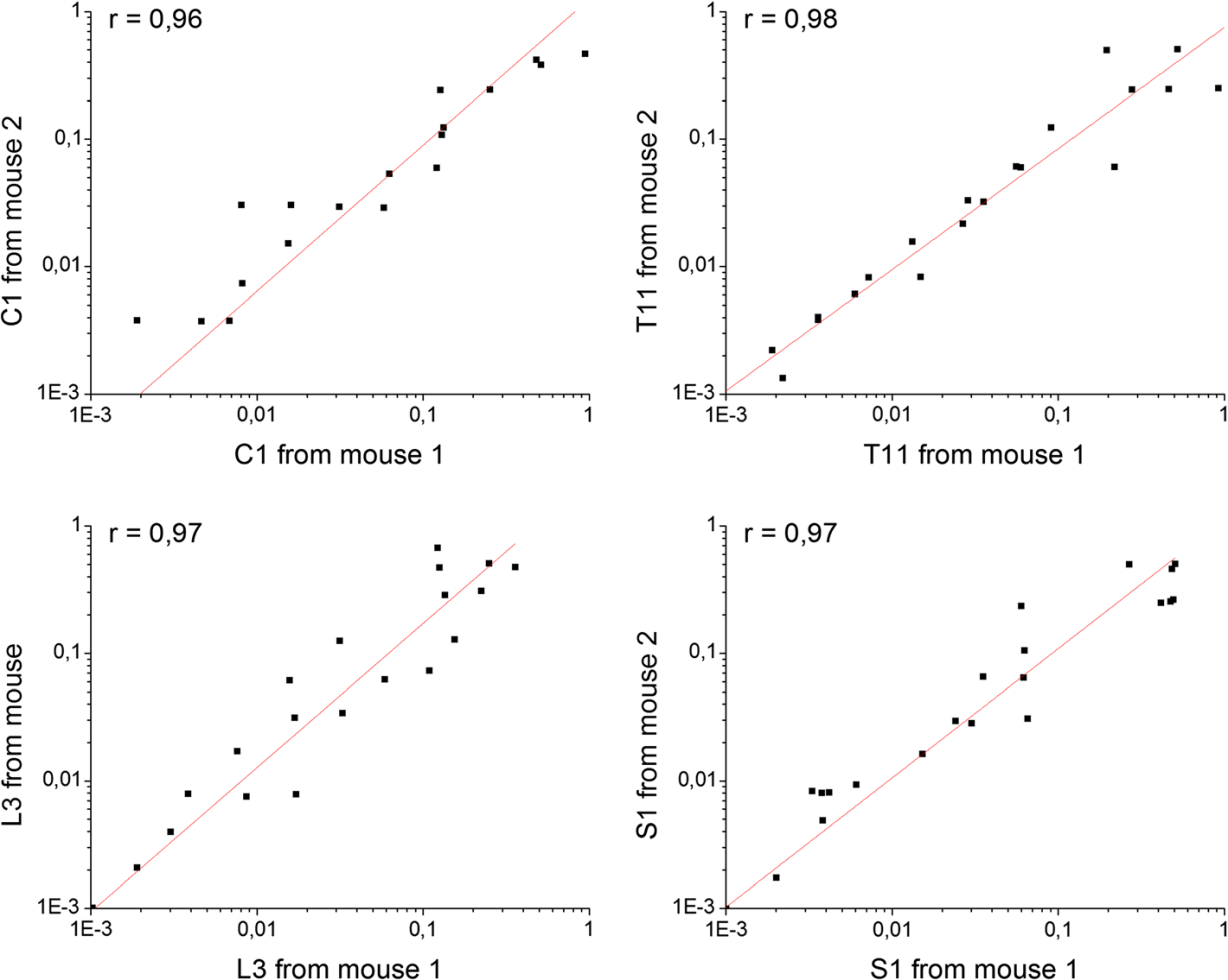
**Correlations between relative mRNA expression in different anatomic segments of two mice. **Examples of comparison between relative mRNA expressions of all tested mouse TRP channel genes in DRGs from segment C1, T11, L3 and S1 between two mice, using the Pearson correlation analysis.

Complementary DNA samples from TG and DRGs, including (from head to tail) 7 cervical segments (C1-C7), 13 thoracic segments (T1-T13), 6 lumbar segments (L1-L6) and first sacral segments (S1) were assayed using the TaqMan assays for all 28 mouse TRP channel genes and phosphoglycerate kinase 1 (PGK1) and hypoxanthine guanine phosphoribosyl transferase 1 (HPRT1) genes as endogenous controls. For each ganglion, qPCR analysis was performed on 3–4 independent biological samples (i.e. independently isolated ganglia from different mice). Pair-wise comparisons of the mRNA expression data of independently isolated and processed ganglia from the same anatomical location displayed a strong correlation, indicating that TRP channel gene expression patterns were relatively constant between samples from different mice (Figure 1).

**Figure 2:**
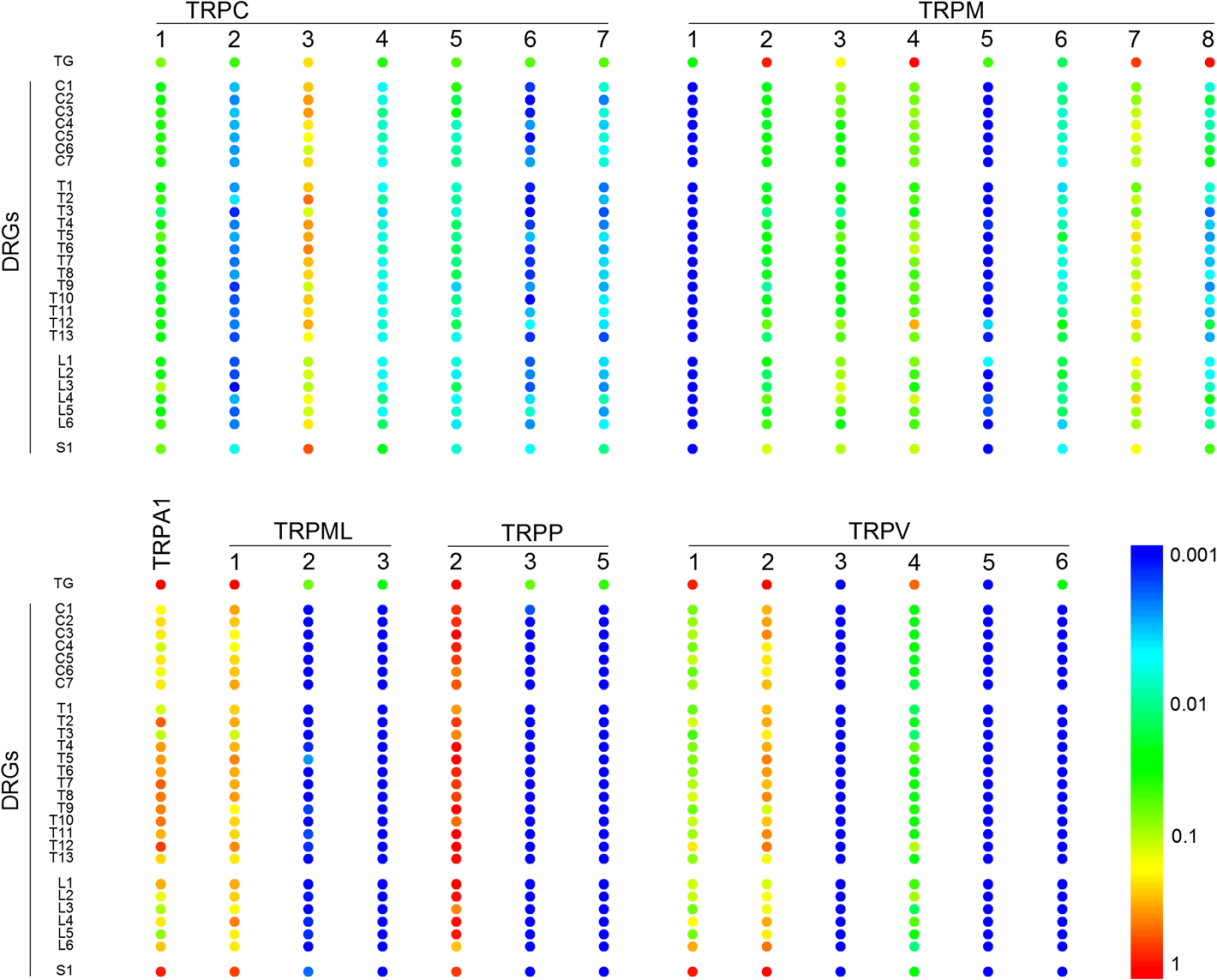
**Expression of TRP mRNAs in each individual segment of the vertebral column. **Comparison of expression levels of TRP channel genes in samples from isolated TG and DRGs (n=3 or 4). The colour code scale represents the average relative expression from all experiments. All corresponding numerical values are deposed in Additional file 2: Table S1. C1-7 – cervical segments; T1-13 – thoracic segments; L1-6 – lumbar segments; S1 – sacral segment.

**Figure 3:**
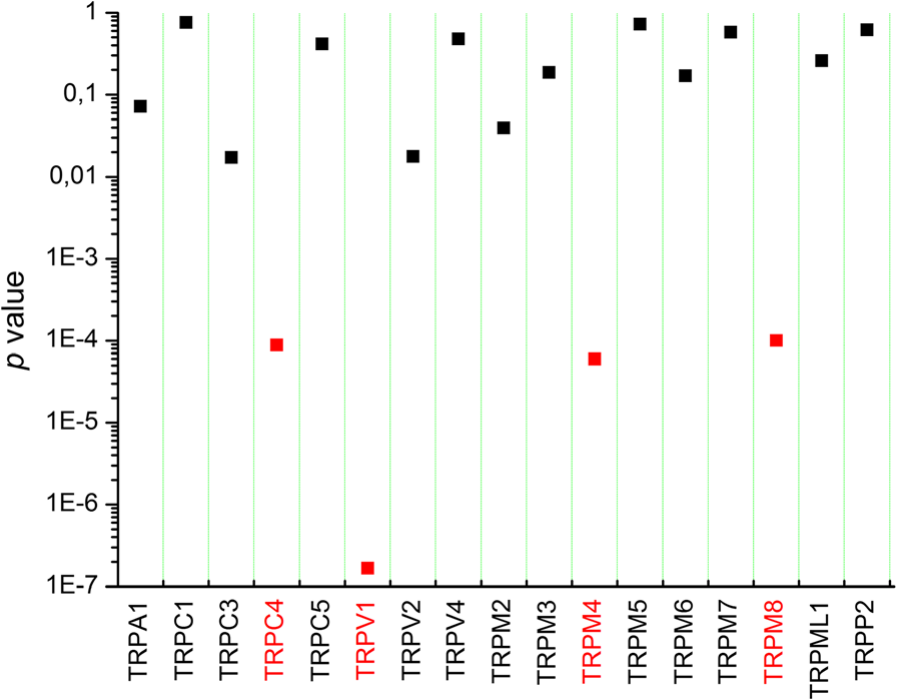
**TRPC4, TRPV1, TRPM4, and TRPM8 display ganglion-to-ganglion variation of mRNA expression in DRGs from distinct vertebral segments. **To demonstrate variable mRNA expression in DRGs all over the vertebral column, ANOVA analysis was performed, using the average relative expression value per segment for every detected TRP gene. Red colour indicates TRP genes showing the most variable mRNA expression in DRGs. *p*: probability.

Figure 2 shows relative expression levels of the different TRP channel genes in individual ganglia from different mice pooled per every anatomic segment (see also Additional file 1: Figure S3 and Additional file 2 Table S1). A set of 11 TRP channel genes, TRPC2, TRPC6, TRPC7, TRPM1, TRPML2, TRPML3, TRPP3, TRPP5, TRPV3, TRPV5 and TRPV6, invariably showed very low (more than 200-fold lower than the level of PGK1) to undetectable levels of mRNA expression in TG and all tested DRGs. For the other 17 TRP channel genes, mRNA was consistently detected in all tested ganglia, albeit at varying expression levels. In particular, the highest expression levels (between 0.1. and 2-fold relative to PGK1) were found for TRPA1, TRPC3, TRPM7, TRPML1, TRPP2 and TRPV2 (Figure 2 and Additional file 2: Table S1). Using one-way ANOVA analysis, we compared relative mRNA expression of all 17 expressed TRP channel genes in DRGs from different segments (Figure 3). Four TRP channels, TRPC4, TRPM4, TRPV1 and TRPM8, displayed significant variations in mRNA expression between ganglia, suggesting that neurons in ganglia from different anatomical segments, innervating distinct parts of the body, might exhibit different sensory profiles and/or TRP channel patterns.

## Discussion

The TRP superfamily is a functionally diverse group of cation channels with widely diverging functional properties. First, there is a striking diversity in the pore permeability of TRP channels, ranging from non-selective cation permeability to high selectivity for divalent cations [14]. Moreover, there is a daunting variety of stimuli that can regulate the gating of the TRP channels, such as physical stimuli (temperature, voltage, mechanical stress), exogenous ligands, intracellular cations and lipid components of the plasma membrane [15]. Several TRP channels, including TRPV1, TRPV4, TRPM3, TRPM8 and TRPA1, are implicated in somatosensation, acting as integral molecular components of the sensory machinery involved in chemo, thermo and/or mechanosensation [16]. Based on experimental data, there was ample evidence for the expression of other TRP channels in sensory neurons, which may either operate as sensors of as yet undefined stimuli or exert other (housekeeping) functions in these cells. The present study had two main aims: (1) to provide a systematic analysis of all TRP channels in different sensory ganglia in mice; and (2) to detect possible ganglion-to-ganglion variations in TRP channel expression. Although destinations of every single DRG neuron projections in mice are still unclear, a homology to human DRG neurons might be instrumental to anticipate TRP channel functions in target organs [17]. In humans, the cervical DRGs mainly projects to dermatomes of the head, upper limbs, hands as well as the eye. The thoracic DRGs innervate dermatomes of the chest and the abdomen (T1-T5 supply the stomach, liver, gallbladder and pancreas while T10-T12 account for the kidneys). The lumbar DRGs innervate dermatomes of the inguinal regions, the anterior and inner surfaces of the lower limbs and feet, while the sacral DRGs project to the remaining posterior and outer surfaces of the lower limbs and lateral margin of the feet (L1-L3 innervate the intestines, L4-S2 are responsible for the innervations of the bladder and prostate, while S3-S5 innervate external genitalia).

We were able to confirm expression of TRP channels that have been previously proposed as sensors in TG and DGR neurons, including TRPA1, TRPV1, TRPV2, TRPV4, TRPM3 and TRPM8 [9,18-22]. Notably, we found that expression levels of TRPV1 and TRPM8 showed statistically significant variation between anatomically different ganglia. For example, TRPM8 expression was significantly lower in the DRG from thoracic segments compared to TG or other regions of the spinal cord, whereas TRPV1 showed significantly higher expression levels in L6 and S1 segments. Such variations may contribute to the variability in functional TRPM8- or TRPV1-mediated responses in neurons isolated from different segments.

Amongst the most highly expressed genes there were also a number of TRP channels for which a function in the sensory system is not yet well established (TRPC3, TRPM7, TRPML1 and TRPP2). TRPC3, together with TRPC6, has recently been proposed as a component of a mechanotransduction complex in sensory neurons [23], although there is little evidence that TRPC3 on its own can form a mechano-gated channel. TRPM7 is a ubiquitously expressed channel that has been implicated in cell proliferation, organ development and intracellular Mg^2+^ homeostasis, although a role in mechanosensation has been suggested in some studies [24,25]. TRPML1 is mainly found in endosomes, and may play a housekeeping role in endosome homeostasis [26]. The function of TRPP2, a TRP channel involved in development of polycystic kidney disease [27], which was found to be the most highly expressed TRP channel gene in the majority of sensory ganglia, is fully unclear.

Expression of 11 TRP channels, TRPC2, TRPC6, TRPC7, TRPM1, TRPML2, TRPML3, TRPP3, TRPP5, TRPV3, TRPV5 and TRPV6, was generally low to undetectable, suggesting that these channels have no specific function in sensory neurons, or only in a very small subset of these cells.

Although these qPCR data provide a valuable comparison of expression at the mRNA level, it is important to remember that mRNA levels not always correlate with the relative abundance of these channels at the protein level or in their functional [28]. Consequently, high mRNA levels do not always indicate that these TRP channels are functionally important, and, vice versa, low mRNA expression levels do not exclude the possibility that these TRP channels are relevant in physiology. Thus, systematic studies using TRP subtype-selective antisera could be useful to extend future studies to the protein level that will further help to assess the role of these channels at the functional level.

## Conclusions

In conclusion, we have developed, compared and refined techniques to quantitatively analyse the relative mRNA expression of all TRP channel genes at the single ganglion level using quantitative real-time PCR. This study also provides for the first time a comparative mRNA distribution profile in TG and DRGs along the entire vertebral column for the mammalian TRP channel family.

## Methods

### Animals

WT C57BL/6J male mice from 10–14 weeks of age were used for all experiments. All experiments approved by the KU Leuven Ethical Committee Laboratory Animals (ECD). Approval was granted under project number P021/2012. The authors’ laboratory has the Belgian Governmental license for small animal experiments (LA1210551).

### RNA extraction and cDNA synthesis

After euthanasia by CO_2_ inhalation, TG and DRGs from each segment of the spine were individually removed, washed in ice-cold PBS, put into the RNA later Stabilization Reagent (Qiagen), immediately snap frozen in liquid nitrogen and kept at −80°C until final processing. Total RNA from TG was extracted using the RNeasy Mini Kit (Qiagen), following manufacturer’s protocol (for all kits in this study only originally supplied components and protocols were used). RNA concentration and quality was assessed using the Experion RNA StdSens Analysis Kit (Bio-Rad), samples with RQI values below 6 were discarded (Additional file 1: Figure S1) [29]. The total RNA from TG (500 ng) subsequently served for cDNA synthesis using Ready-To-Go You-Prime First-Strand Beads (GE Healthcare). The total RNA from DRGs (10 ng) was extracted using the RNeasy Plus Micro Kit (Qiagen). RNA concentration and quality was assessed using the Experion RNA HighSens Analysis Kit (Bio-Rad), samples with RQI values below 6 were discarded (Additional file 1: Figure S1). The RNA subsequently served for cDNA synthesis using the SuperScript VILO cDNA Synthesis Kit (Life Technologies). Generated cDNAs were stored at −20°C (for the MIQE guidelines checklist see Additional file 3).

### RNA preamplification

cDNA from DRGs was subjected to the TaqMan PreAmp Master Mix Kit (Life Technologies). This kit is intended for use with very small quantities of cDNA (1–250 ng) and increases the quantity of specific cDNA targets for gene expression analysis using TaqMan gene expression assays (Table 1). Preamplification uniformity was assessed using the ΔΔCt method, which involves a qPCR with both the original cDNA and the preamplified cDNA as a starting template. A ΔΔCt value close to zero indicates that there is preamplification uniformity, with limits set at ± 1.5. All target genes produced ΔΔCt values within these limits (Additional file 1: Figure S2 B,C), indicating that there is no significant amplification bias.

**Table 1:** List of TaqMan gene expression assays (life technologies) used in the study

**Gene Name**	**Assay ID**	**GenBank mRNA**	**Exon boundary**	**Assay location**	**Amplicon length**
TRPA1	Mm00625268	AY231177.1	7 - 8	942	73
TRPC1	Mm00441975	AF191551.1	8 - 9	1546	129
TRPC2	Mm00441984	AF111107.1	15 - 16	2675	77
TRPC3	Mm00444690	AF190645.1	3 - 4	1086	129
TRPC4	Mm00444284	AF011543.1	6 - 7	1934	122
TRPC5	Mm00437183	AF029983.1	5 - 6	1755	105
TRPC6	Mm01176083	U49069.1	1 - 2	463	65
TRPC7	Mm00442606	AF139923.1	6 - 7	1678	93
TRPM1	Mm00450619	AF047714.1	6 - 7	822	90
TRPM2	Mm00663098	AB166747.1	29 - 30	4265	107
TRPM3	Mm00616485	AK051867.1	21 - 22	3135	75
TRPM4	Mm00613173	AJ575814.1	9 - 10	1204	78
TRPM5	Mm00498453	AB039952.1	18 - 19	2787	73
TRPM6	Mm00463112	AK080899.1	13 - 14	1520	125
TRPM7	Mm00457998	AY032951.1	13 - 14	1752	125
TRPM8	Mm00454566	AF481480.2	24 - 25	3389	89
TRPML1	Mm00522550	BC005651.1	2 - 3	316	64
TRPML2	Mm00509841	AF503575.1	1 - 2	163	68
TRPML3	Mm00460328	AF475086.1	8 - 9	1095	82
TRPP2	Mm00435829	AF014010.1	1 - 2	673	84
TRPP3	Mm00619572	AF271381.1	4 - 5	870	74
TRPP5	Mm00450423	AF182033.1	6 - 7	1022	96
TRPV1	Mm01246302	AJ620495.1	9 - 10	1593	68
TRPV2	Mm00449223	AB021665.1	3 - 4	643	95
TRPV3	Mm00454996	AF510316.1	1 - 2	42	57
TRPV4	Mm00499025	AJ296078.1	2 - 3	596	56
TRPV5	Mm01166037	AK085479.1	8 - 9	1214	93
TRPV6	Mm00499069	AB037373.1	1 - 2	325	63
PGK1	Mm00435617	M15668.1	5 - 6	546	137
HPRT1	Mm00446968	J00423.1	6 - 7	570	65

### Quantitative real-time PCR

qPCR reactions (20 μl), composed of 2 μl cDNA template, Universal TaqMan MasterMix (2x concentrated, Life Technologies), TaqMan assay (20x concentrated, Life Technologies) and H_2_O, were performed with the 7500 Fast Real-Time PCR System (Life Technologies). Reactions, ran in triplicate, were incubated at 50°C for 2 min and 95°C for 10 min, followed by 40 cycles of 95°C for 15 sec and 60°C for 1 min. Non-template controls (NTCs) were used as negative controls in every experiment. PGK1 and HPRT1, selected using the geNorm application [30], were used as endogenous controls (see also Additional file 4: Table S2). Data represent relative expression of detected mRNA normalized to PGK1 mRNA, which was used as a calibrator for comparative analysis [31].

### Statistical analysis

Origin software (version 8.6; OriginLab) was used for statistical analysis and data display. Data are represented as mean ± SEM. One-way ANOVA was used for statistical comparison between groups.

## Abbreviations

TG: Trigeminal ganglia; DRG: Dorsal root ganglia; TRP: Transient receptor potential; mRNA: Messenger RNA; RQI: RNA quality indicator; PGK1: Phosphoglycerate kinase 1; HPRT1: Hypoxanthine guanine phosphoribosyl transferase 1.

## Competing interests

The authors declare that they have no competing interests.

## Authors’ contributions

IV, GO and TV designed research; IV performed research and analyzed data; IV, GO and TV wrote the paper. All authors read and approved the final manuscript.

## Supplementary Material

Additional file 1: Figure S1 Examples of quality control measurements of total RNA extractions from different DRGs. Figure S2. Optimization of cDNA libraries for quantitative PCR analysis. Figure S3. Expression of TRP mRNAs in each individual segment of the vertebral column. Click here for file

Additional file 2: Table S1 Relative expression levels of the different TRP channel genes in individual ganglia. Expression levels of different mice were pooled per segment and expressed as mean ± SEM. C1-7 – DRGs in cervical segments; T1-13 – DRGs in thoracic segments; L1-6 – DRGs in lumbar segments; S1 – DRG in sacral segment. Click here for file

Additional file 3 MIQE guidelines checklist. Click here for file

Additional file 4: Table S2 Ct values for the reference mRNA genes of individual DRGs per segment. The mean was calculated of experiments done in triplicate. Click here for file
