# Supplementary material for: Systematic and quantitative mRNA expression analysis of TRP channel genes at the single trigeminal and dorsal root ganglion level in mouse
Source: BMC Neurosci. 2013 Feb 14;14:21. doi: 10.1186/1471-2202-14-21 (PMC3576292; doi:10.1186/1471-2202-14-21)

Supplementary data

Supplementary Figure 1 – Examples of quality control measurements of total RNA extractions from different DRGs.

1 µl of total RNA from 4 different DRG preparations (A-D) was assayed using the Experion electrophoretic station following the manufacturer's protocol (Bio-Rad). Electrophoresis gel-like picture and 4 electropherograms for every total RNA sample are presented. L: standard RNA ladder; RQI: RNA quality indicator.

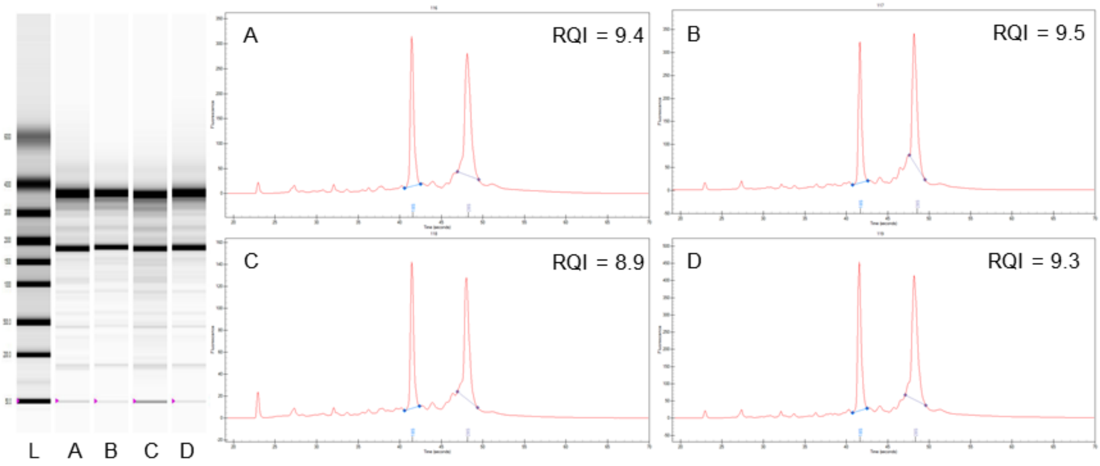

Supplementary Figure 2 - Optimization of cDNA libraries for quantitative PCR analysis. (A) The Ready-To-Go You-Prime First-Strand Beads (A; GE Healthcare), the High-Capacity cDNA Reverse Transcription Kit (B; Life Technologies), and SuperScript VILO cDNA Synthesis Kit (C; Life Technologies) was used for generation of cDNA libraries using the same amount of total RNA. Efficiencies of used cDNA synthesis kits were compared using quantitative PCR with GAPDH and β-actin TaqMan assays. The lowest raw Ct values correspond to the most efficient cDNA synthesis process. (B) cDNA samples were preamplified with selected TRP specific TaqMan gene expression assays and TaqMan PreAmp Master Mix following the manufacturer’s protocol (Life Technologies). The graph represents the correlation between Ct values of qPCR data obtained with or without preamplification of cDNA used as a template. (C) Uniformity of preamplified cDNA libraries from (B) was tested using qPCR and randomly selected TRP specific TaqMan assays (one for each TRP subfamily) following the PreAmp manufacturer’s protocol (Life Technologies). The dashed line represents the limits for the acceptable amplification bias using the PreAmp protocol (Life Technologies).

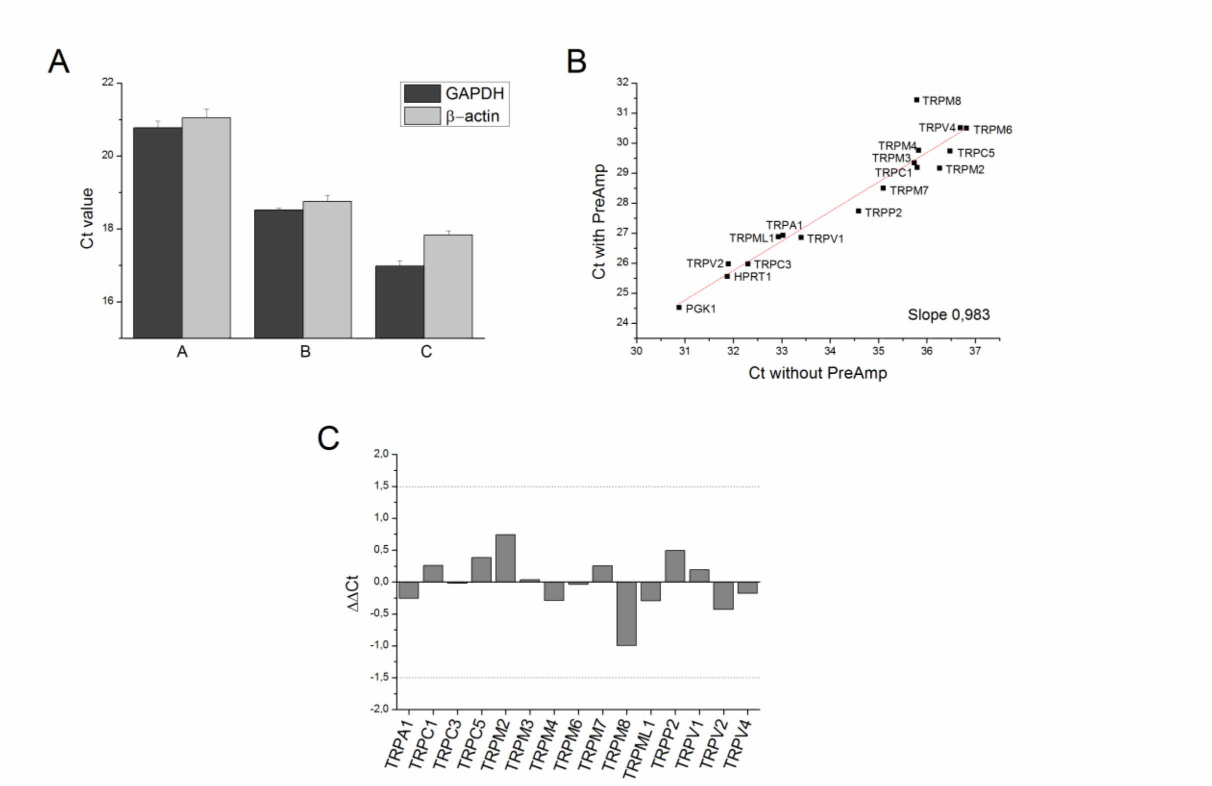

Supplementary Figure 3 – Expression of TRP mRNAs in each individual segment of the vertebral column.

Comparison of expression levels of TRP channel genes in samples from isolated DRGs (n=3 or 4). All corresponding numerical values are deposed in Supplementary Table 1. C1-7 – cervical segments; T1-13 – thoracic segments; L1-6 – lumbar segments; S1 – sacral segment.

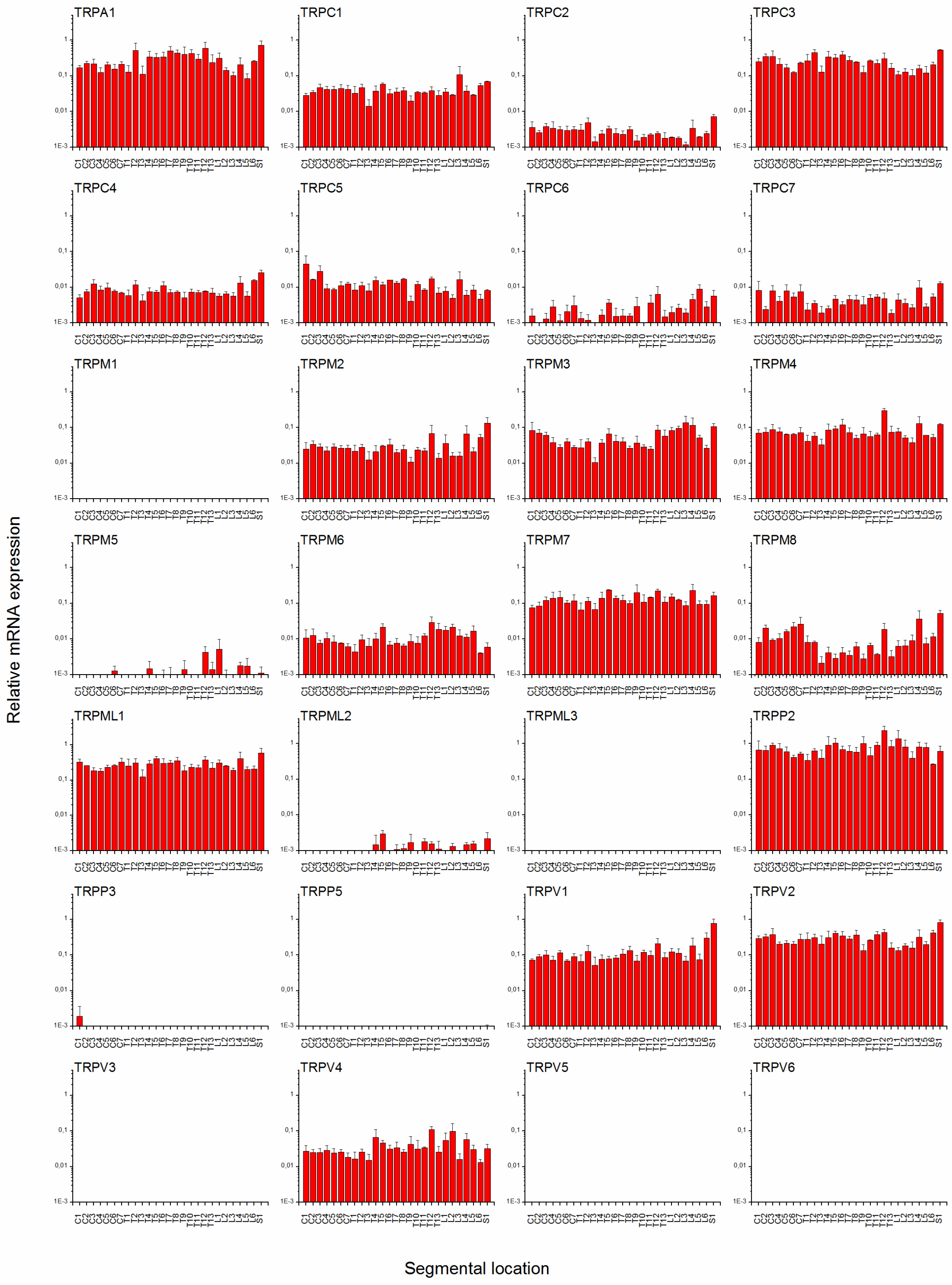

Supplement: Additional file 1: Figure S1 — Examples of quality control measurements of total RNA extractions from different DRGs. Figure S2. Optimization of cDNA libraries for quantitative PCR analysis. Figure S3. Expression of TRP mRNAs in each individual segment of the vertebral column. [file 1471-2202-14-21-S1.pdf]
